# Peritoneal carcinomatosis with desmoplasia and osseous metaplasia mimicking encapsulating peritoneal sclerosis in a cat: case report

**DOI:** 10.3389/fvets.2023.1298736

**Published:** 2023-12-01

**Authors:** So-Jeong Nam, Sun-Hye Song, Seung-Hyun Lee, So-Young Jeung, Jae Gon Ah, Su-Hyung Lee, Min-Ok Ryu

**Affiliations:** ^1^VIP Animal Medical Center KR, Seoul, Republic of Korea; ^2^Section of Surgical Sciences, Epithelial Biology Center, Vanderbilt University Medical Center, Nashville, TN, United States; ^3^Laboratory of Internal Medicine, Department of Veterinary Clinical Science, College of Veterinary Medicine, Seoul National University, Seoul, Republic of Korea

**Keywords:** encapsulated peritoneal sclerosis, peritoneal carcinomatosis, calcification, feline, case report

## Abstract

A 13-year-old neutered male Korean short-hair cat presented with anorexia, lethargy, and a severely distended abdomen, suggestive of ascites. Abdominocentesis yielded serosanguineous fluid. A subsequent diagnostic workup, including blood tests, ascitic fluid analysis, imaging studies [radiography, ultrasound, and computed tomography (CT)], and histopathological examination, was performed to identify the underlying cause. Imaging studies revealed characteristics of encapsulating peritoneal sclerosis (EPS) such as peritoneal thickening, fat stranding, and calcification. During laparotomy, fibrous membranes encapsulating the abdominal organs and ascites were observed, and multiple calcified regions were detected on the abdominal wall. Histopathological analysis confirmed the diagnosis of poorly differentiated invasive malignant neoplasms, which were further classified as carcinomatosis based on positive cytokeratin and negative vimentin immunohistochemistry results. To our knowledge, this is the first report of sclerosing peritoneal carcinomatosis with osseous metaplasia in a cat.

## 1 Introduction

Encapsulating peritoneal sclerosis (EPS), also called sclerosing encapsulating peritonitis (SEP), is a rare condition characterized by a thickened fibrous peritoneum encasing the abdominal organs and first described in human medicine (1), often presenting with ascites. In human medicine, peritoneal dialysis induced EPS is most common and abdominal surgery or foreign body can cause EPS (2). Idiopathic EPS is also reported in some cases in human medicine (2). In veterinary medicine, EPS is categorized as either primary (idiopathic) or secondary (3, 4) as in human. In felines, EPS cases involving idiopathic EPS, infection, or leiomyosarcoma have been reported (3–5). On imaging, EPS presents as abdominal distention, mesenteric fat stranding, loculated abdominal fluid, gathered bowel loops and calcification both in human medicine (6) and veterinary medicine (5).

Here, we present the case of a 13-year-old neutered male Korean short-hair cat in which EPS was suspected based on radiography, ultrasound, and computed tomography (CT); however, histopathology revealed an unexpected diagnosis of sclerosing peritoneal carcinomatosis (SPC) with osseous metaplasia. Given the challenging nature of EPS diagnosis and its potential therapeutic implications, we aimed to explore the diagnostic process and highlight the importance of considering SPC with osseous metaplasia as a differential diagnosis, when EPS is suspected based on imaging findings. This case report provides valuable clinical insights, underscoring the significance of histopathological analysis for establishing an accurate diagnosis and guiding the appropriate management of complicated cases.

## 2 Case description

A 13-year-old neutered male Korean short-hair cat presented with anorexia and lethargy that had been ongoing for at least 2 weeks. The cat was kept indoors and had never been taken for walks outdoors. Basic feline vaccinations (feline herpesvirus-1, feline calicivirus, feline parvovirus, *Chlamydia psittaci*) were administered during its young-adult stage. While deworming was performed annually, preventive measures against ectoparasites were not applied. There was no history of trauma. The cat had only undergone surgery twice, including neutering when it was under 1 year old and wart removal 2 years ago. The wart, located on the instep of the right forelimb, measured less than 0.5 cm in diameter, was encapsulated, and not grown for several years, suggesting it was considered a benign tumor.

The cat appeared depressed, but responsive upon presentation. Physical examination revealed normal vital signs, except for a severely distended abdomen that exhibited fluctuations suggestive of ascites. Abdominocentesis was performed, resulting in the removal of 1 liter of serosanguineous fluid. Blood work and fluid analyses were subsequently performed to identify the underlying causes of the ascites.

The blood test results were all within the normal range, except for a mildly elevated serum symmetric dimethylarginine level of 21 μg/dL (reference range: 0–14 μg/dL). The NT-proBNP level was below 50 pmol/L (reference range: 0–270 pmol/L), ruling out a cardiogenic etiology for the ascites. Additionally, the feline leukemia virus (FeLV) antigen, feline immunodeficiency virus (FIV) antibody, and heartworm antigen test results using SNAP Feline Triple Test kit (IDEXX laboratories, Westbrook, ME, United States) were negative. In the ascitic fluid analysis, the total nucleated cell count (TNCC) was 2040 cells/μL, and the total protein (TP) was 3.4 g/dL, confirming the ascites as a modified transudate. In cytology, the ascitic fluid contained small numbers of macrophages, neutrophils, lymphocytes, and red blood cells (RBCs), without any evidence of neoplastic cells. Furthermore, PCR testing for feline coronavirus and microbial culture of the ascitic fluid yielded negative results, indicating a low possibility of feline infectious peritonitis (FIP) or septic peritonitis as etiologies.

**Figure 1:**
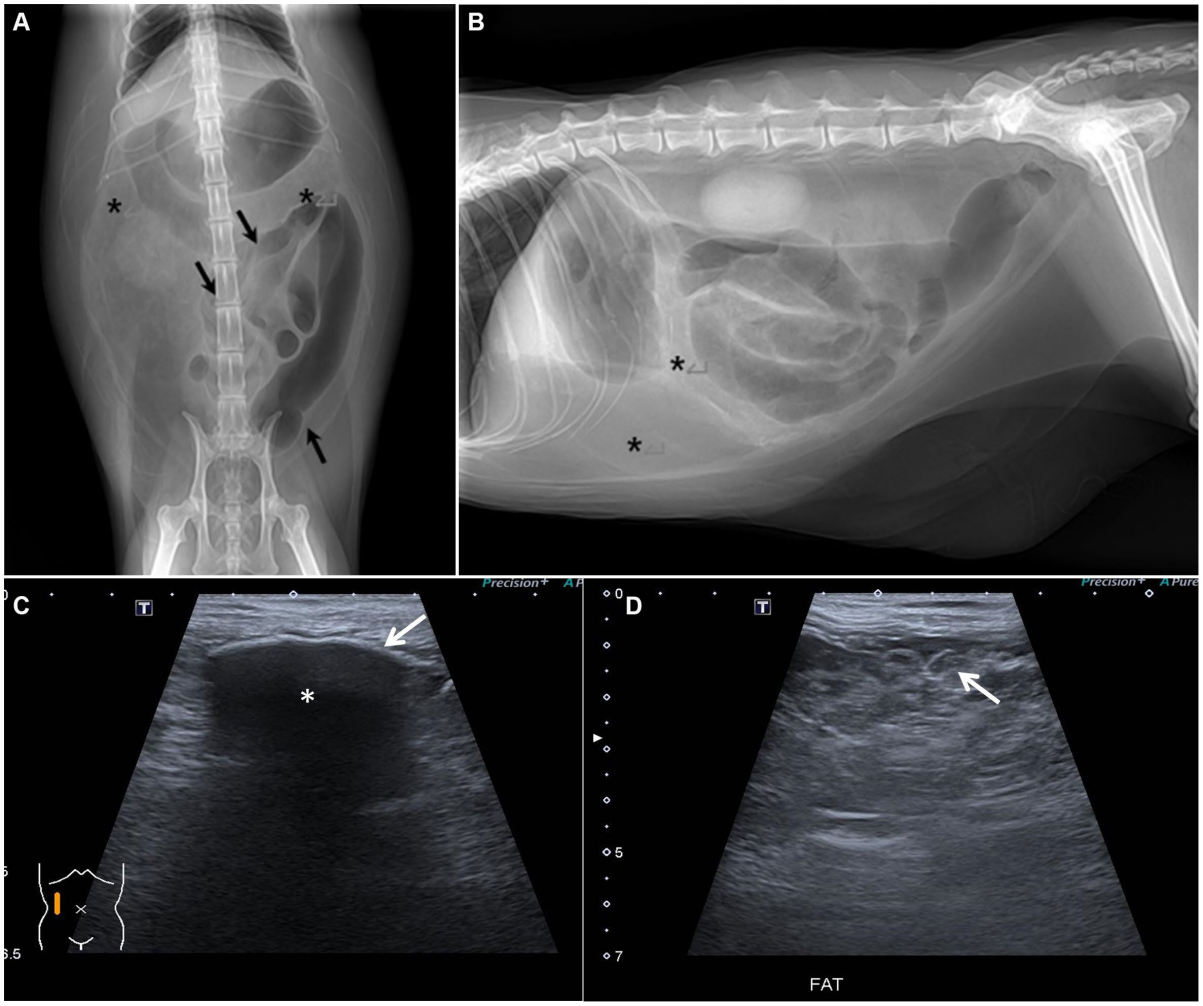
Abdominal radiographs and ultrasound. **(A,B)** Abdominal radiographs after abdominocentesis. **(A)** Ventro-dorsal image. **(B)** Right lateral image. The gas-filled bowel is displaced and gathered leftward (black arrow). The opacity assumed to be omental and mesenteric fat is heterogeneously increased (asterisks). **(C,D)** Abdominal ultrasound. **(C)** Peritoneal calcification was suspected due to hyperechoic peritoneal change (white arrow) with strong acoustic shadowing (asterisk). **(D)** Peritoneal fat was multilobulated and showed heterogeneously high echogenicity (white arrow).

Radiographs following abdominocentesis revealed an abnormally heterogeneously high fat opacity in the upper and right mid-lower abdominal areas (Figure 1). Furthermore, the gas-filled intestines were displaced into the left abdomen. Abdominal ultrasonography revealed a multilobulated intestinal mesentery and omentum with focally increased fat echogenicity and a small amount of free fluid in the abdominal cavity (Figure 1). Additionally, some of the abdominal parietal peritonea were thickened, and the mesenteric peritoneum of the right abdomen was focally calcified, showing a hyperechoic lining with strong acoustic shadowing. The gas-filled intestinal segments were mildly corrugated.

The cat received fluid therapy, antibiotic treatment, and H2 blocker in accordance with colitis management. Additionally, to address the issue of anorexia, a nasoesophageal tube was inserted for enteral feeding.

**Figure 2:**
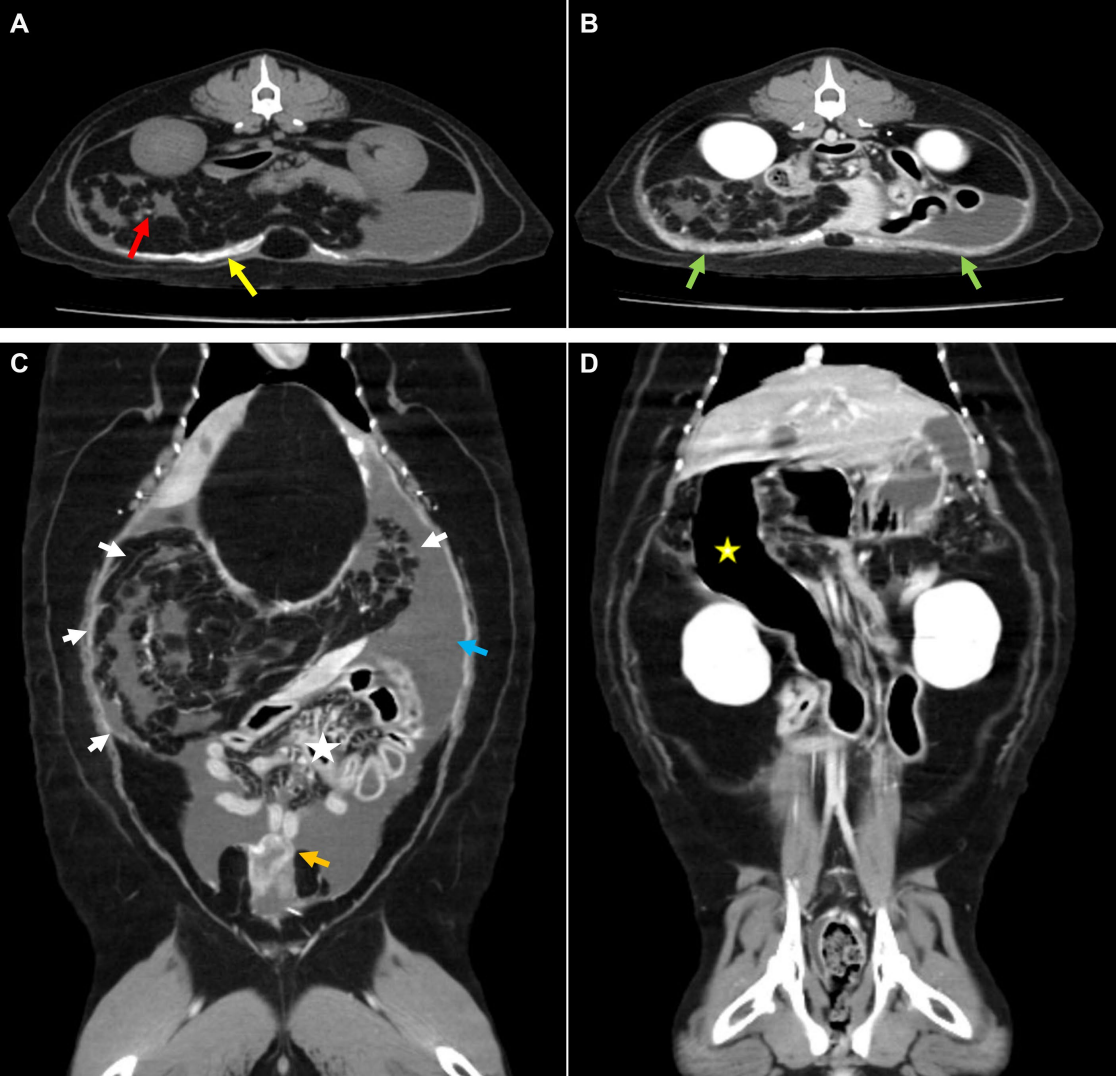
Computed tomography. **(A)** Pre-contrast and **(B–D)** post-contrast CT images. **(A)** Broad calcification at the parietal peritoneum (yellow arrow) and small calcified foci at the mesentery (red arrow). **(B)** Thickened peritoneum showed strong enhancement (green arrow). **(C)** Gathered omentum and mesentery at the craniolateral abdominal cavity were observed with fat stranding (white arrow), which caused intestinal displacement toward the lower abdomen (asterisk). Surrounding peritoneal effusion (blue arrow) and thickened visceral peritoneum adjacent to the urinary bladder (orange arrow) were also detected. **(D)** An abnormally dilated descending duodenum with gas was identified (yellow asterisk).

Computed tomography (CT; Brivo CT 385, GE Hangwei Medical System Co., Ltd., Beijing, China) was performed under general anesthesia in sternal recumbency on next day to achieve an accurate diagnosis and for surgical planning (Figure 2). For CT scans, general anesthesia was performed for a total of 1 h, and major vital signs were measured at 5 min intervals. Butorphanol 0.2 mg/kg and midazolam 0.1 mg/kg were used as pre-medication, and induction was performed using propofol 6 mg/kg IV and isoflurane was used for maintenance. Acquisition parameter were as follows: helical mode, 120kVp, exposure 120–200 mAs, 2.5 mm slice thickness, 2.5 mm spacing. Contrast injection was manually performed and images were acquired 35 s following bolus injection. Multi-planar reformatting (MPR) was performed to obtain transverse, sagittal, and dorsal planar images with following parameters; 2 mm slice thickness, 2 mm spacing. Mesenteric and omental fat stranding was observed as multiple linear hyper-attenuations clumped in the right craniolateral abdominal cavity. This resulted in left caudolateral displacement of the stacked bowels and peritoneal effusion. Some small bowels were filled with gas, and the descending duodenum was severely dilated with retained gastric fluid. Strong enhancement was observed in the thickened parietal and visceral peritoneum around the urinary bladder. Extensive calcification was observed in the ventral parietal peritoneum and small calcified foci were observed in the omentum and mesentery. Based on these CT findings, EPS was suspected to have caused the ascites.

After CT examination, elevated temperature (body temperature 40°C) was noted on regular vital check, and normalized after physical cooling. Fluid therapy, antibiotics, antiemetics, proton pump inhibitor, appetite stimulator, and pain medication (butorphanol 0.2 mg/kg q6h) was administered during hospitalization.

**Figure 3:**
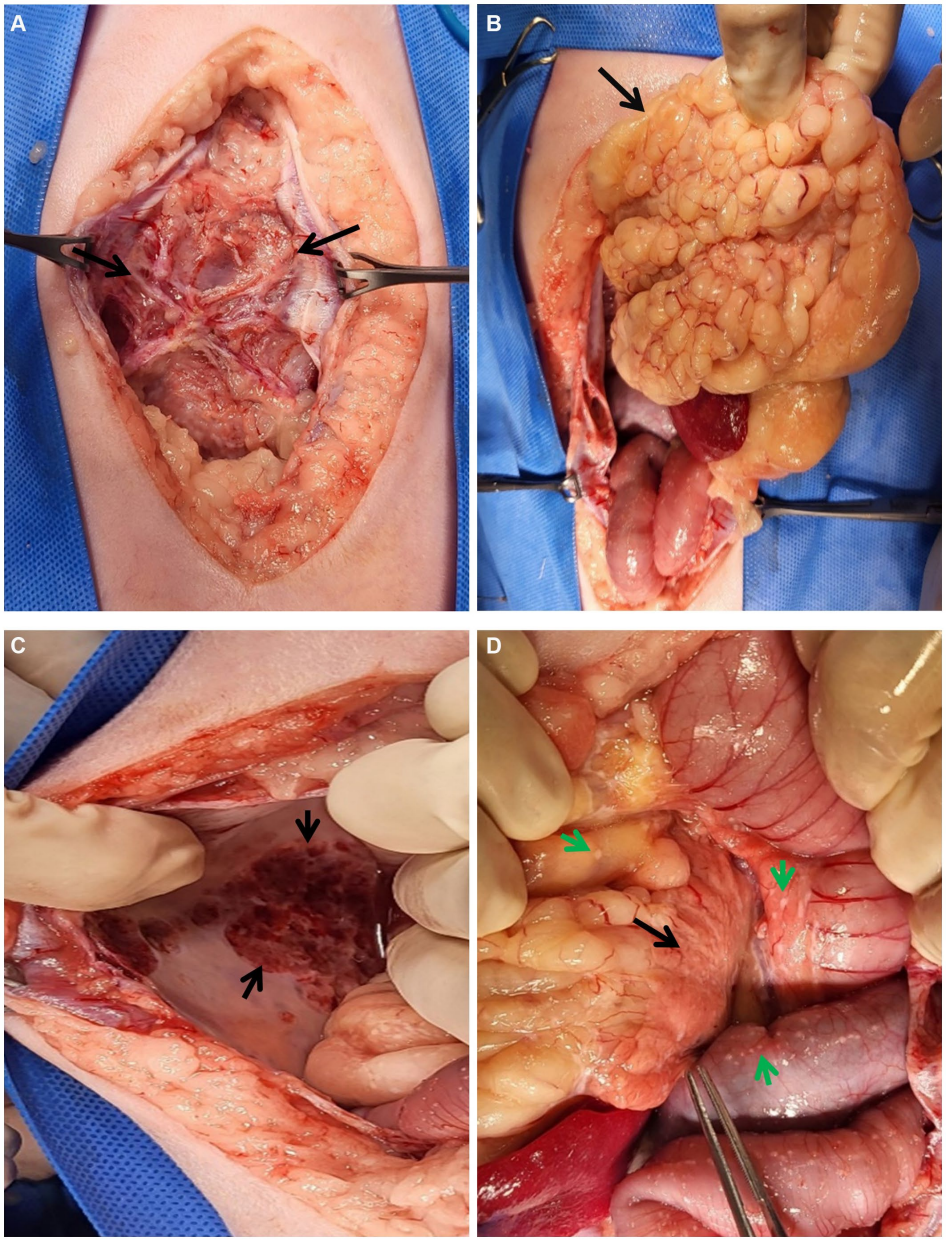
Gross lesions during laparotomy. **(A–D)** Gross lesions of the abdominal organs during laparotomy. **(A)** An unusual fibrous membrane (black arrows) covers the abdominal organs and fluids. After incision of the membrane, **(B)** the omentum (black arrow) looked abnormally lobulated and cranially condensed. **(C)** The parietal peritoneal wall was calcified (black arrow). **(D)** Multiple fibrous plaques (green arrows) were observed throughout the peritoneum, especially in the intestines and abdomen. The pancreas (black arrow) also appeared to be atrophied.

Two days after CT examination, to determine the cause of the suspected EPS, multiple biopsies were performed via laparotomy. Cefazolin 30 mg/kg IV and hydromorphone 0.1 mg/kg IV was used as pre-medication for laparotomy and propofol 6 mg/kg IV was used as induction agent. General anesthesia was performed for a total 90 min from induction to endotracheal tube extubation, major vital signs were measured at 5 min intervals. Isoflurane was used for anesthesia maintenance and depth was adjusted upon procedure’s type and patient’s vital sign. Upon incision of the abdominal wall, a fibrous membrane was observed connecting the wall to the encapsulated abdominal organs and ascitic fluid (Figure 3). Additionally, omental fat was calcified and condensed in the cranial direction. The parietal portion of the abdominal wall also exhibited signs of calcification, which prompted the collection of biopsy samples from these areas. Abnormal nodules of various sizes were diffusely distributed in the peritoneum and intestinal serosal wall. Gross examination further revealed an erythematous bladder serosal wall and a contracted pancreas, consistent with the CT findings.

**Figure 4:**
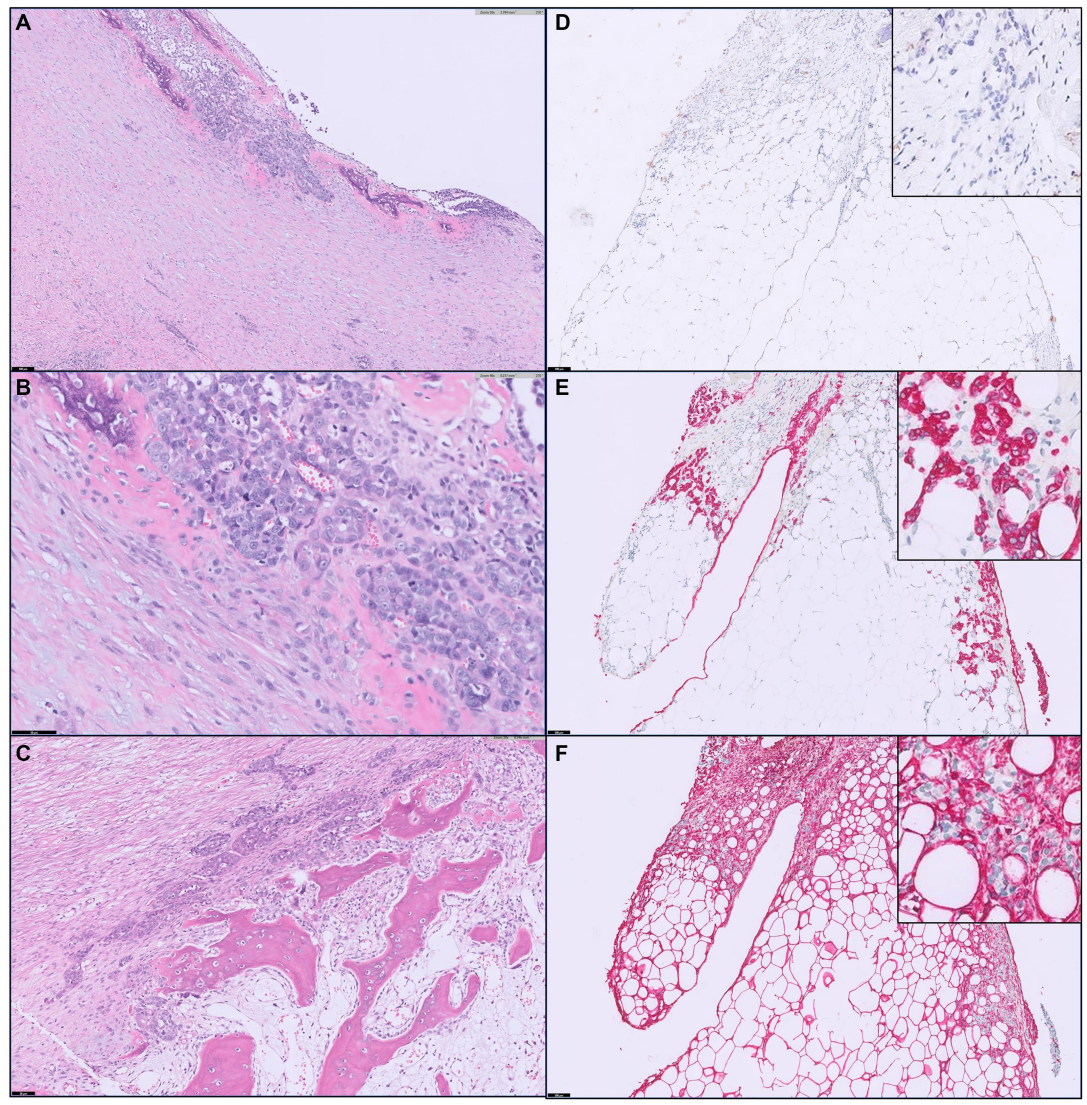
Histopathological features of the biopsy samples. **(A,B)** Neoplastic cells form small nests or islands, surrounded by thick fibrosis with basophilic mineralization or mucinous inter or intracellular matrix. The neoplastic cells are characterized by a moderate degree of cellular atypia, a high nuclear-cytoplasmic ratio, prominent nucleoli, and an increased mitotic index. Scale bar = 100 μm for **(A)** or 50 μm for **(B)**. **(C)** Bone trabeculae are present at the center of calcified abdominal wall tissue sample, close to neoplastic cell clusters. A number of spindle-shaped stromal cells and red blood cells are interspersed between bone trabeculae. Scale bar = 50 μm. **(D–F)** Immunohistochemistry staining for WT1 **(D)**, cytokeratin **(E)** and vimentin **(F)** on neoplastic cells within the omental fat is presented. Insets revealed cancer cells with a negative WT1 expression **(D)**, while the typical positive result of WT1 should exhibit brownish nuclear staining. Insets demonstrated that cytokeratin-positive cancer cells (red, **E**) are completely negative for vimentin **(F)**, which is strongly expressed in the stromal cells surrounding cancer cells. Cytokeratin and vimentin staining are observed in the cytoplasm, indicated by the presence of red-colored particles, signifying positivity. Scale bar = 100 μm.

Biopsy samples obtained from the omentum, calcified abdominal walls, and fibrous tissues attached to the abdominal walls were submitted for histopathological examination at IDEXX Laboratories (Westbrook, ME, United States). Hematoxylin and eosin (H&E) staining revealed poorly differentiated, invasive malignant neoplasms (mitotic count 5–8 in 10 high powered 0.237 mm^2^ fields) (Figures 4A–C). The neoplastic cells had indistinct cell borders, a small to moderate amount of eosinophilic cytoplasm, round nuclei with finely stippled chromatin, and one or two distinct nucleoli. The fibrous tissues attached to the abdominal wall were composed of adipose connective tissue and abundant scirrhous stroma with islands and clusters of poorly differentiated neoplastic cells. In sections of the omentum and calcified abdominal wall, bone formation characterized by bone trabeculae and cells within lacunae was prominently observed in close proximity to neoplastic cells.

Immunohistochemistry (IHC) was conducted at IDEXX Laboratories to categorize neoplastic cell types. Cytokeratin (AE1/AE3) and vimentin were used as stains to further classify the tumor type as carcinoma, sarcoma, or mesothelioma. A diagnosis of mesothelioma typically involves positivity for both cytokeratin and vimentin markers. Conversely, if neoplastic cells are positive for only cytokeratin, it suggests carcinoma, while positivity for only vimentin indicates sarcoma. The results revealed that over 90% of invasive, poorly differentiated neoplastic cells exhibited strong immunoreactivity to cytokeratin (AE1/AE3) in the cytoplasm (Figure 4E). However, only a few cells within the neoplastic cell population tested positive for vimentin (Figure 4F), aligning with the characteristics of carcinoma. Additionally, immunostaing for Wilm’s tumor 1 (WT1) was performed to definitively exclude mesothelioma, and the result was negative (Figure 4D).

As no suspicious primary tumor was observed during ultrasound, CT scans, or laparotomy, the cat was diagnosed with peritoneal carcinomatosis. Furthermore, taking into consideration the presence of fibrous tissue encapsulating abdominal organs and bone formation, a diagnosis of SPC with osseous metaplasia was established. Unfortunately, due to the rapid deterioration of the patient following laparotomy, euthanasia was ultimately performed, and no treatment trial was initiated. Euthanasia was carried out by administration of T-61™ (Intervet International GmbH, Germany) at a dose of 0.5 mL/kg after anesthetization with a propofol IV bolus of 6 mg/kg. A post-mortem examination could not be conducted, as it was declined by the owner.

## 3 Discussion

This case report highlights the diagnostic challenges encountered in a feline patient presenting with ascites and suspected EPS based on imaging characteristics. Its clinical significance lies in the unexpected histopathological confirmation of SPC with osseous metaplasia, emphasizing the importance of considering SPC as a differential diagnosis when EPS is suspected on imaging.

In humans, distinctive EPS findings on CT include peritoneal thickening, loculated fluid collection, peritoneal calcification, bowel tethering or matting, peritoneal enhancement, and occasional bowel wall thickening (6). Some studies have suggested that the CT appearance, along with clinical symptoms, may allow for a confident diagnosis of EPS, reducing the necessity of histopathological confirmation (6–8).

Limited information is available on the imaging features of EPS in veterinary medicine. A study of animals with presumptive EPS included seven dogs and two cats, all of which presented with ascites (5). Radiographic characteristics in this study included the gathering of bowel loops and abdominal distention, while ultrasonographic findings showed echogenic fluid, gathered or corrugated bowel loops, and enlarged abdominal lymph nodes. All animal patients who underwent CT presented with mesenteric fat stranding. Other feline case reports also showed similar imaging features of corrugated small intestinal loops, dilated stomachs or intestines filled with fluid, enlarged lymph nodes on ultrasound, and an ascites-filled distended abdomen on radiographs (3, 9).

In our case, the cat exhibited imaging features suggestive of EPS, including a one-sided stacked intestine on radiography, and a multilobulated intestinal mesentery and omentum with increased fat echogenicity, peritoneal thickening with calcification, and corrugated intestines on ultrasonography. Additionally, the CT findings revealed mesenteric and omental fat stranding, one-sided stacked bowels, gas-filled bowels, peritoneal thickening, and calcification. Based on these combined imaging findings, the patient was presumptively diagnosed with EPS. During laparotomy, we observed a fibrous membrane encapsulating the abdominal organs and ascites, similar to the previously reported gross features of another cat with EPS (5).

Histopathological examination and IHC of the biopsy samples were crucial for providing a definitive diagnosis, revealing poorly differentiated, invasive malignant neoplasms that were positive for cytokeratin (AE1/AE3) and negative for vimentin, and therefore consistent with carcinoma. In this study, no suspected primary tumors were identified during CT or ultrasound examinations, nor were they detected on visual inspection during laparotomy. Therefore, this case was diagnosed with peritoneal carcinomatosis, desmoplasia, and osseous metaplasia. To the best of our knowledge, there have been no reported cases of peritoneal carcinomatosis with osseous metaplasia, calcification, or mineralization in the veterinary literature to date. In two retrospective studies of the imaging features of feline peritoneal carcinomatosis, none of the 24 cats examined showed mineralization on radiographs, ultrasound, or CT scans (10, 11). This report presents the first documented case of peritoneal carcinomatosis with calcification or EPS in a cat.

The etiology of desmoplasia and osseous metaplasia in peritoneal carcinomatosis is presumed to be similar to the mechanisms by which desmoplasia and osseous metaplasia occur in carcinoma. The pathogenesis of desmoplasia in carcinoma involves complex interactions between neoplastic cells and the tumor microenvironment, which includes a significant role played by cancer-associated fibroblasts (CAFs) (12, 13). CAFs secrete extracellular matrix (ECM) proteins, such as collagen and fibronectin, and signaling molecules that promote tissue remodeling and angiogenesis, which can facilitate tumor growth and invasiveness (12). In human medicine, severity of desmoplasia was demonstrated to be related to negative prognosis in numerous carcinoma types including pancreatic ductal adenocarcinoma, colorectal carcinoma, and uterine endometrioid adenocarcinoma (14–17). In this feline case, given the pronounced scirrhous change, it is likely associated with the rapid deterioration of the patient’s condition. In human medicine, some studies showed that metastatic cancers had significant levels of desmoplasia comparable to that seen in primary tumors including pancreatic ductal adenocarcinoma and colorectal cancer (14–16). The substantial desmoplasia observed in this feline case report of peritoneal carcinomatosis is considered to be similar in mechanism to desmoplasia in metastatic lesions.

Bone formation in tumor can be categorized in three types; osteogenesis in osteosarcoma, osteoblastic differentiation in sarcomatoid carcinoma, and osseous metaplasia (18). Carcinoma with osseous metaplasia has been reported in oral squamous cell carcinoma, gastrointestinal carcinoma, and thymic lymphosarcoma in felines (19–22). While there is no established mechanism for osseous metaplasia in any type of carcinoma in feline patients, mucin, osteopontin, MAPK, P53, and CD44 produced by tumor cells are thought to be involved in osseous metaplasia in human intestinal carcinoma (23, 24) and bone morphogenetic proteins (BMP) induced transformation of stromal cells into osteoblasts, alkaline-phosphatase activity, local calcium and phosphate levels are thought to be involved in osseous metaplasia in human urothelial carcinoma (18). Given the extensive desmoplasia observed in this case, it is plausible to consider the possibility of osseous metaplasia resulting from the transformation of stromal cells. Further research examining the mechanisms related to osseous metaplasia in peritoneal carcinomatosis in cats would be valuable.

In conclusion, this case report offers valuable clinical insights into the diagnostic process for a feline patient with suspected EPS, where histopathological analysis revealed an unexpected diagnosis of SPC. Veterinarians should consider SPC as part of the differential diagnosis when encountering cases of suspected EPS, especially when radiological and clinical features are atypical or inconclusive. The sharing of similar clinical experience will improve diagnostic accuracy and potentially enhance therapeutic approaches for feline peritoneal diseases.

## Data availability statement

The original contributions presented in the study are included in the article/supplementary material, further inquiries can be directed to the corresponding author.

## Ethics statement

Written informed consent was obtained from the owner for the publication of this case report.

## Author contributions

S-JN: Writing – original draft, Conceptualization, Investigation. S-HS: Formal analysis, Writing – original draft. Se-HL: Investigation, Writing – review & editing. S-YJ: Investigation, Writing – review & editing. JA: Project administration, Writing – review & editing. Su-HL: Formal analysis, Writing – original draft, Writing – review & editing. M-OR: Conceptualization, Writing – original draft, Writing – review & editing.

## References

[1] Danford CJ Lin SC Smith MP Wolf JL. Encapsulating peritoneal sclerosis. World J Gastroenterol. (2018) 24:3101–11. doi: 10.3748/wjg.v24.i28.3101, PMID: 30065556 PMC6064970

[2] Machado NO. Sclerosing encapsulating peritonitis: review. Sultan Qaboos Univ Med J. (2016) 16:e142–51. doi: 10.18295/squmj.2016.16.02.003, PMID: 27226904 PMC4868512

[3] Kiniger C Janssen JN Lederer KA Lipnik K Doulidis PG. Sclerosing encapsulating peritonitis in cats: a two-case report and literature review. JFMS Open Rep. (2023) 9:20551169231178447. doi: 10.1177/20551169231178447, PMID: 37434990 PMC10331345

[4] Hardie EM Rottman JB Levy JK. Sclerosing encapsulating peritonitis in four dogs and a cat. Vet Surg. (1994) 23:107–14. doi: 10.1111/j.1532-950x.1994.tb00454.x, PMID: 8191669

[5] Gremillet BCH Porsmoguer C Bolen G Billen F Noël S Brutinel F. Imaging findings in dogs and cats with presumptive sclerosing encapsulating peritonitis. Front Vet Sci. (2022) 9:891492. doi: 10.3389/fvets.2022.891492, PMID: 35754547 PMC9218854

[6] George C Al-Zwae K Nair S Cast JEI. Computed tomography appearances of sclerosing encapsulating peritonitis. Clin Radiol. (2007) 62:732–7. doi: 10.1016/j.crad.2007.01.022, PMID: 17604760

[7] Tannoury JN Abboud BN. Idiopathic sclerosing encapsulating peritonitis: abdominal cocoon. World J Gastroenterol. (2012) 18:1999–2004. doi: 10.3748/wjg.v18.i17.1999, PMID: 22563185 PMC3342596

[8] Zhang Z Zhang M Li L. Sclerosing encapsulating peritonitis: three case reports and review of the literature. J Int Med Res. (2020) 48:300060520949104. doi: 10.1177/0300060520949104, PMID: 32811273 PMC7441290

[9] Sonck L Chiers K Ducatelle R Van Brantegem L. Encapsulating peritoneal sclerosis in a young cat. Vet Rec Case Rep. (2018) 6:e000541. doi: 10.1136/vetreccr-2017-000541

[10] Wu B Kastl B Cino-Ozuna AG Springer NL Thakkar R Biller D. Feline sarcomatoid renal cell carcinoma with peritoneal carcinomatosis and effusion. J Vet Diagn Investig. (2022) 34:153–9. doi: 10.1177/10406387211054826, PMID: 34713776 PMC8689035

[11] Weston PJ Baines SJ Finotello R Mortier JR. Clinical, CT, and ultrasonographic features of canine and feline pleural and peritoneal carcinomatosis and sarcomatosis. Vet Radiol Ultrasound. (2021) 62:331–41. doi: 10.1111/vru.12951, PMID: 33476083

[12] Franco OE Shaw AK Strand DW Hayward SW. Cancer associated fibroblasts in cancer pathogenesis. Semin Cell Dev Biol. (2010) 21:33–9. doi: 10.1016/j.semcdb.2009.10.010, PMID: 19896548 PMC2823834

[13] Lee JI Campbell JS. Role of desmoplasia in cholangiocarcinoma and hepatocellular carcinoma. J Hepatol. (2014) 61:432–4. doi: 10.1016/j.jhep.2014.04.014 24751832

[14] Ao T Kajiwara Y Yonemura K Shinto E Mochizuki S Okamoto K. Morphological consistency of desmoplastic reactions between the primary colorectal cancer lesion and associated metastatic lesions. Virchows Arch. (2020) 477:47–55. doi: 10.1007/s00428-019-02742-2, PMID: 31932918

[15] Whatcott CJ Diep CH Jiang P Watanabe A LoBello J Sima C. Desmoplasia in primary tumors and metastatic lesions of pancreatic Cancer. Clin Cancer Res. (2015) 21:3561–8. doi: 10.1158/1078-0432.Ccr-14-1051, PMID: 25695692 PMC4526394

[16] Nakayama H Ohuchida K Yoshida M Miyazaki T Takesue S Abe T. Degree of desmoplasia in metastatic lymph node lesions is associated with lesion size and poor prognosis in pancreatic cancer patients. Oncol Lett. (2017) 14:3141–7. doi: 10.3892/ol.2017.6549, PMID: 28927058 PMC5588067

[17] Wei S Conner MG Zhang K Siegal GP Novak L. Juxtatumoral stromal reactions in uterine endometrioid adenocarcinoma and their prognostic significance. Int J Gynecol Pathol. (2010) 29:562–7. doi: 10.1097/PGP.0b013e3181e36321, PMID: 20881855

[18] Razafimahefa J Gosset C Mongiat-Artus P Andriamampionona TF Verine J. Stromal osseous metaplasia in urothelial carcinoma of the bladder: a rare case report and literature review. Diagn Pathol. (2019) 14:75. doi: 10.1186/s13000-019-0851-z, PMID: 31299983 PMC6626396

[19] Jung JH Kim NY Yang Y Seo D Choi G Hong H. Metastatic intestinal adenocarcinoma with osseous metaplasia in two domestic Korean shorthair cats. J Vet Sci. (2023) 24:24. doi: 10.4142/jvs.23124, PMID: 37638712 PMC10556286

[20] Uneyama M Chambers JK Nakashima K Uchida K Nakayama H. Histological classification and immunohistochemical study of feline colorectal epithelial tumors. Vet Pathol. (2021) 58:305–14. doi: 10.1177/0300985820974279, PMID: 33208031

[21] Martin CK Tannehill-Gregg SH Wolfe TD Rosol TJ. Bone-invasive oral squamous cell carcinoma in cats: pathology and expression of parathyroid hormone-related protein. Vet Pathol. (2011) 48:302–12. doi: 10.1177/0300985810384414, PMID: 20940448 PMC4519039

[22] Thilsted JP Bolton RG. Thymic lymphosarcoma with bony metaplasia in a cat. Vet Pathol. (1985) 22:424–5. doi: 10.1177/030098588502200422, PMID: 3839950

[23] Haque S Eisen RN West AB. Heterotopic bone formation in the gastrointestinal tract. Arch Pathol Lab Med. (1996) 120:666–70. PMID: 8757473

[24] Liu X Xu J Chen L. Colorectal carcinoma with osseous metaplasia. Oncotarget. (2017) 8:65407–13. doi: 10.18632/oncotarget.18577, PMID: 29029440 PMC5630340

